# Early Detection of Ototoxicity Using Serial Mobile Audiometry, Otoacoustic Emissions Testing, and Inner Ear Biomarker Measurement in Patients Receiving Platinum-Based Chemotherapy Treatment: It is Feasible to Implement in a National Health Service (NHS) Cancer Ambulatory Care Setting

**DOI:** 10.1097/MAO.0000000000004856

**Published:** 2026-02-25

**Authors:** Nishchay Mehta, Asma Awad, Marina Forbes, Eleftheria Iliadou, Roulla Katiri, Tanjinah Ferdous, Helen Lowe, Leah Ensell, Constantine Alifrangis, Anne G.M. Schilder, Rachael Windsor

**Affiliations:** a National Institute for Health Research, University College London Hospitals Biomedical Research Centre, London, UK; b Ear Institute, University College London; c Royal National ENT & Eastman Dental Hospitals, University College London Hospitals NHS Foundation Trust; d Experimental Cancer Medicine Centre Good Clinical Laboratory Practice Facility, Cancer Institute, University College London; e Division of Cancer; f Children and Young People’s Cancer Service, University College London Hospitals NHS Foundation Trust, London, UK

**Keywords:** Audiological monitoring, High-frequency audiometry, Otoacoustic emissions, Otolin, Ototoxicity, Platinum-based chemotherapy, Prestin, Serum biomarkers

## Abstract

**Importance:**

Platinum-based chemotherapy is widely used in the management of solid tumors, and up to 60% of patients will experience ototoxic side effects, including hearing loss and tinnitus. The advent of novel otoprotective drugs generates a need for more flexible and innovative real-time approaches to drug-induced ototoxicity monitoring in cancer patients.

**Objective:**

To assess the feasibility of recruitment and compliance to monitoring of ototoxicity with mobile out-of-booth audiometry, Distortion Product Otoacoustic Emission (DPOAE) testing, and serum biomarker measurements.

**Design:**

Prospective observational study.

**Setting:**

Cancer ambulatory care.

**Participants:**

Patients undergoing platinum-based chemotherapy for bone or testicular cancer.

**Results:**

Twenty-three patients were enrolled between February 2021 and June 2023. The mean age was 17.5 years (range: 13 to 36 y). Average compliance to monitoring assessments was 86.5% for audiometry, 84.8% for DPOAEs, and 84.3% for serum biomarker measurements. End-of-treatment audiometric changes indicative of ototoxicity were found in 18 (78.2%) of the participants.

**Conclusions and relevance:**

High compliance with serial mobile audiometry and otoacoustic emissions testing, and serum biomarker measurement suggests that ototoxicity monitoring can be successfully performed in cancer ambulatory care rather than in an audiology department setting. Further investigation is required to understand the role of serum prestin and otolin-1 in monitoring ototoxicity.

## Introduction

Platinum-based chemotherapy, most commonly with cisplatin and carboplatin, is widely used in a variety of cancers^1^. Ototoxicity is a common side effect of these drugs^2,3^, with up to 60% of patients developing irreversible hearing loss^1,4^, tinnitus^5^, and balance^6^ problems. These side effects adversely affect the quality of life in patients dealing with cancer, both during and after treatment^7,8^. Oncological management of chemotherapy-induced ototoxicity includes dose reduction or substitution of platinum-based drugs with less ototoxic chemotherapy, both of which may compromise the likelihood of cure.

In-booth audiometry within a specialist audiology department often presents practical challenges for patients undergoing active cancer treatment, particularly for those with reduced mobility or who are hospitalized for chemotherapy-induced toxicity. This may lead to delayed diagnosis and missed opportunity for early detection and timely intervention^9^. Tablet-based out-of-booth audiometry in a quiet room has been recently validated with high sensitivity and specificity when compared with audiologist-led manual testing in a soundbooth^10^, and review of the technology suggests it may have applications in ototoxicity monitoring^11^.

Emerging evidence suggests sensorineural hearing loss may be associated with changes in peripheral blood levels of the inner ear proteins prestin and otolin-1^12–14^. With several novel otoprotective drugs in the pipeline^15–17^, the first approved for clinical use^18–20^, there is an urgent need for new approaches to real-time ototoxicity monitoring in patients undergoing platinum-based chemotherapy.

This study explored the feasibility of monitoring ototoxicity in a cancer ambulatory care setting using serial mobile out-of-booth audiometry and otoacoustic emissions testing. We also report exploratory observations of changes in serum prestin and otolin-1 biomarker levels in patients receiving platinum-based chemotherapy for bone and testicular cancer.

The primary objective was:

- recruitment and compliance with the study protocol.

Secondary objectives were:

- pure-tone air-conduction thresholds at 0.25 to 16 kHz using out-of-booth tablet-based audiometry at baseline and before and after each chemotherapy treatment cycle,
- DPOAE amplitudes at 1.5, 2, 3, 4, 6, and 8 kHz using mobile equipment at baseline and before and after each chemotherapy treatment cycle,
- hearing- and tinnitus-related quality of life at baseline and end-of platinum-based chemotherapy treatment (final study visit), and
- brief exploration of serum prestin and otolin-1 levels as potential biomarkers through treatment.

## Materials and methods

This prospective observational study was approved by the London Camberwell St Giles Research Ethics Committee and Health and Research Authority (IRAS: 271954, CPMS: 44553). Bone sarcoma patients were recruited from the London Sarcoma Service at University College London Hospital (UCLH). Testicular cancer patients were recruited from the St Bartholomew’s Germ Cell Referral Centre. We aimed to include a pragmatically chosen number of 20 patients at each site. This estimate was based on available guidance^21^, taking into account the study timeframe and referral numbers in the months before the study start, aiming to gain insights into implementation challenges.

Patient inclusion criteria included age 10 to 50 years, diagnosis of bone sarcoma or testicular cancer, and approval for platinum-based chemotherapy treatment, and a history of normal hearing and ability to provide informed consent or assent. Exclusion criteria were prior platinum-based chemotherapy within 5 years, self-reported pre-existing hearing loss, inability to self-administer tablet-based audiometry, signs of active outer or middle ear disease, and inability to understand spoken and written English.

Eligible patients were identified by the oncologist and provided with study information sheets at least 24 hours before they were due to commence their first chemotherapy cycle. Informed consent and assent were taken by the treating oncologist after all questions had been answered.

Patients with high-grade osteosarcoma were treated with standard MAP (methotrexate, doxorubicin, and cisplatin) chemotherapy as per EURAMOS-1 recommendations^22^. Treatment comprised two 5-week preoperative cycles of cisplatin 40 mg/m^2^, day 1, 2, and 3 (P), doxorubicin 75 mg/m^2^ (A), and 2 consecutive doses of methotrexate 12 g/m^2^ (M) followed by 4 postoperative cycles, 2 MAP and 2 MA (maximum anticipated cisplatin dose 480 mg/m^2^). One patient was treated with carboplatin and etoposide chemotherapy for recurrent Ewing sarcoma as per the rEECur clinical trial^23^. Their treatment comprised carboplatin 400 mg/m^2^ (day 1) and etoposide 120 mg/m^2^, days 1, 2, and 3 to a maximum of 6 cycles.

Patients with testicular cancer were treated with standard BEP (bleomycin, etoposide, and cisplatin) chemotherapy as per the European Society for Medical Oncology (ESMO) clinical guidelines^24^, or with carboplatin AUC10 as per local policy for International Germ Cell Cancer Collaborative Group (IGCCCG) good prognosis metastatic seminoma. BEP comprised bleomycin 30,000 IU D1, 8, 15, etoposide 165 mg/m^2^ D1-3, cisplatin 50 mg/m^2^ D1, D2 on a 21-day cycle for 3 cycles for good prognosis disease. Patients with good prognosis seminoma received carboplatin AUC10 IV^25^ for 3 cycles.

Data were collected from all participants at baseline, and before and after each chemotherapy cycle throughout their platinum-based treatment regimen. The schedule of assessments was tailored to the chemotherapy regimen, and a case report form (CRF) was developed for each participant, including all scheduled chemotherapy visits and planned study visits and procedures. These visits were scheduled as follows: within 2 to 3 weeks from baseline to the start of chemotherapy treatment, within 2 days before or after each chemotherapy cycle, and within 2 to 4 weeks after completion of the last chemotherapy cycle (eFigure 1, Supplemental Digital Content 1, http://links.lww.com/MAO/C371 in the supplement, for schedule of assessments). If there was a deviation from the chemotherapy schedule and hence the study visits and procedures, or if planned study visits or procedures were canceled or missed, the reasons were recorded as free text on the CRF.

The research audiologist conducted all study procedures in the quietest room available in the cancer ambulatory care unit. Ambient sound pressure levels were measured using the tablet-based audiometer, aiming for a maximum permissible level of 40 dB A averaged across 0.5 to 8 kHz, as per the manufacturer’s recommendations^26^. When ambient noise levels exceeded 40 dB A, testing continued, but a note of the level was made on the participant’s CRF. Out of the 95 test sessions conducted in total, the ambient noise levels exceeded recommendations in 23 (24.2%), with a median of 43.4 dB A (range: 40.2 to 52.1 dB A).

The researcher recorded whether the participant reported any hearing or balance symptoms, the duration of each assessment, and any observations or patient comments regarding the study procedures. Assessments included otoscopic examination with a handheld otoscope, tablet-based pure-tone audiometry, DPOAEs, and blood collection for serum prestin and otolin-1 levels analysis. Pure-tone audiometry was conducted with the Shoebox Pro portable audiometer (SHOEBOX Ltd)^26^ using circum-aural RadioEar DD450 sound-attenuating headphones. Pure-tone air-conduction thresholds were measured at frequencies ranging from 0.25 to 16 kHz. For frequencies 0.25 to 8 kHz, the research audiologist established thresholds following the British Society of Audiology (BSA) recommended procedures^27^. Thresholds at 10 to 16 kHz were measured through the automated self-administered function on the Shoebox Pro portable audiometer. Participants watched the tutorial on the Shoebox Pro tablet before starting their testing, and the research audiologist supported them when needed. When participants were too unwell to self-administer testing at 10 to 16 kHz, the audiologist measured these thresholds manually according to BSA recommended procedures^27^. DPOAE testing was conducted with the Otodynamics 10FLX-S Otoport Flexi OAE (Otodynamics Limited)^28^ at 1.5 to 8 kHz frequency range using half-octave resolution and medium noise rejection. The intensities of f1 and f2 were 65 and 55 dB SPL, respectively, and the f2/f1 ratio was 1.22. The evoked responses for 2f1–f2 were assessed.

Hearing-related quality of life questionnaires were completed before starting and at completion of platinum chemotherapy. For participants aged between 10 and 12 years, the Hearing Environments and Reflection on Quality of Life (HEAR-QL-26) questionnaire^29^ was used, and for children and adolescents aged 13 to 18 years, the HEAR-QL-28 questionnaire^30^. For patients aged 18 years and above, the Hearing Handicap Inventory for Adults (HHIA) questionnaire^31^ was completed. When participants reported tinnitus during their treatment, the Tinnitus Functional Index (TFI) questionnaire^32^ was completed.

For the exploration of serum prestin and otolin-1 levels as potential biomarkers through treatment, samples were collected from indwelling central venous lines of the bone sarcoma patients during scheduled routine chemotherapy visits coinciding with scheduled study visits. We did not take these samples from testicular cancer patients for operational reasons. A Vacutainer Serum-Separating Tube was used to collect 5 mL of blood, which was centrifuged at room temperature (18 to 21 °C) for 20 minutes at 1000*g* 30 minutes after collection. Samples were stored in a −70 °C or lower freezer until the time of assaying. Prestin and otolin-1 concentrations were quantified using the manufacturer’s instruction manual from the ELISA kits (purchased from MyBioSource, Prestin: Catalog No: MBS2890542, Human Otolin-1: Catalog No: MBS9312960). Each sample and standard were run in triplicate. The coefficient of variation of the kits was <15% intra- and inter-assay precision. The Spectramax i3 plate reader was used for absorbance reading. Test results were shown as pg/mL. The standard curve range was 31.25 to 1000 pg/mL.

### Statistical analysis

R^33^ was used for data visualization and statistical analysis. Descriptive statistics were used to summarize sample demographics, including age distribution, sex ratio, and treatment regimen. Recruitment and compliance with the study protocol were expressed as percentages based on the number of specific tests completed over the number of specific tests scheduled. For the purposes of this subgroup analysis, ototoxicity was defined as per the ASHA guidelines^34^

- an increase in hearing threshold of ≥20 dB at a single frequency, or
- a change of ≥10 dB at 2 or more adjacent frequencies, or
- a loss of responses at 3 consecutive frequencies, where responses were previously obtained.

To explore our hypothesis that baseline serum prestin and otolin-1 levels would be altered in relation to PTA threshold shifts, we observed these biomarker changes in participants with ototoxicity and those with no documented ototoxicity separately.

## Results

**Figure 1:**
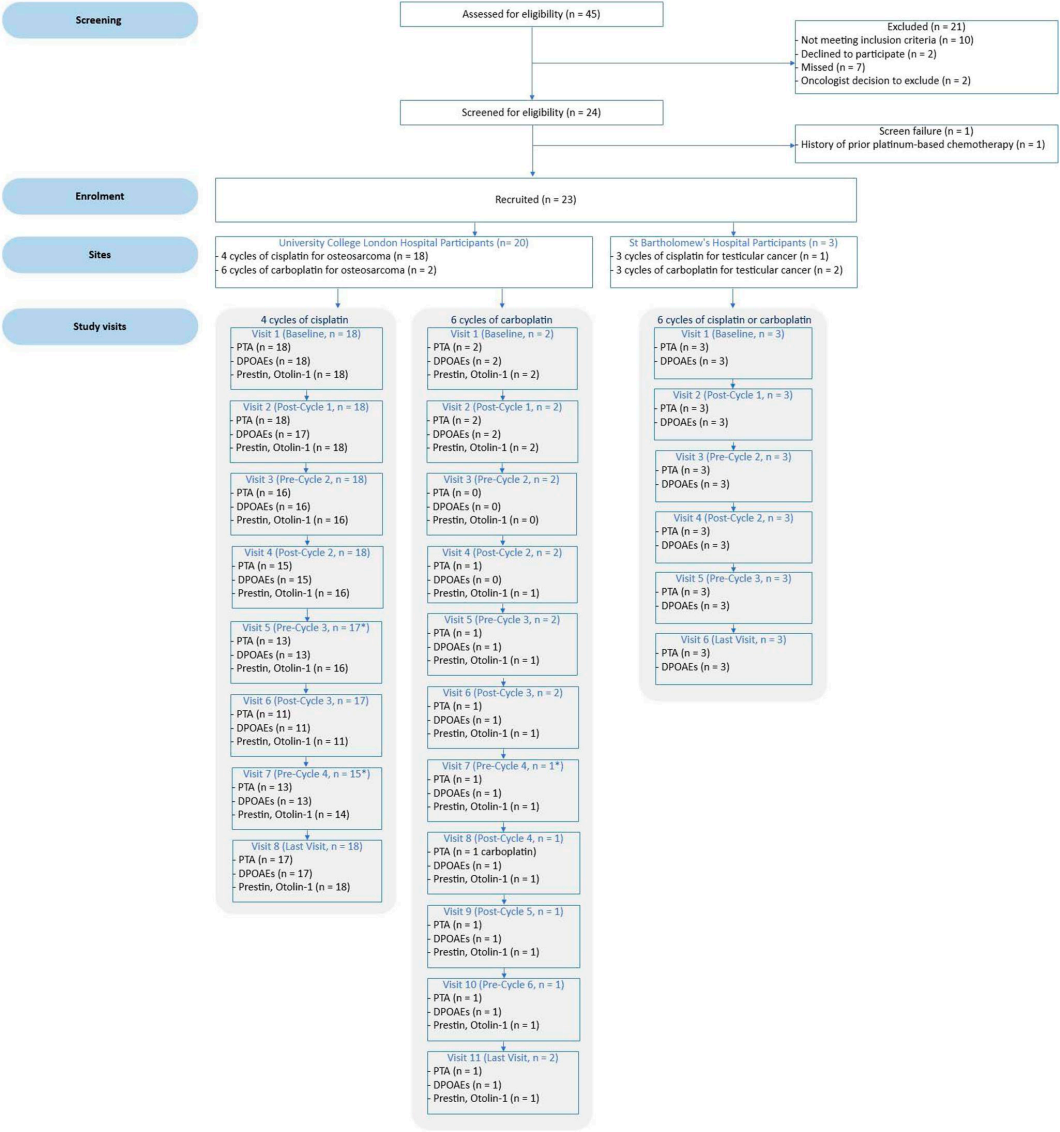
Flow diagram summarizing the participants’ study and assessments completed.

Between February 2021 and June 2023, 45 patients were screened for eligibility. Of those, 11 (24.4%) did not meet the inclusion criteria, 7 (15.5%) were not included due to the research audiologist being unavailable for the baseline measurements, 2 (4.4%) declined participation, and 2 (4.4%) were too unwell to be approached for participation in the study (Fig. 1). Twenty-three participants were recruited (Table 1). Four (17.4%) of the 23 participants reported no hearing-related symptoms throughout their chemotherapy treatment. Three (13.0%) reported tinnitus only, 6 (26.1%) hearing issues only, and 8 (34.8%) co-existing tinnitus and hearing issues. There was no information available for 2 participants. Median cisplatin dose was 480 mg/m^2^ (range: 240 to 480). Seven (36.8%) participants had cisplatin omitted from their last cycle due to hearing loss and/or tinnitus.

**Table 1:** Recruited participant demographic and disease-specific characteristics

Bone Sarcoma Participants (n=20, 87%)	Sex (M/F)	Age Range in Years (Mean)	Baseline PTA_0.5-4 kHz_ (dB HL) (Right/Left Ear)	Baseline EHF-PTA_10-16 kHz_ (dB HL) (Right/Left Ear)
Cisplatin (n=18)	(16/2)	13-22 (17)	9/9	15.6/13.4
Carboplatin (n=2)	(2/0)	18-22 (20)	11/15.5	10.8/10
Testicular cancer participants (n=3, 13%)				
Cisplatin (n=1)	(1/0)	36 (36)	5/8	23/26
Carboplatin (n=2)	(2/0)	28-30 (29)	9.5/10.5	20.8/30
Total (n=23)	(21/2)	13-36 (19)	9.6/11	15.9/17

EHF indicates extended high frequency, F, Female, M, male; PTA, pure-tone audiometry.

A summary of the schedule of assessments, including screening and enrollment of eligible participants, and procedures conducted at each study visit, can be found in eFigure 1, Supplemental Digital Content 1, http://links.lww.com/MAO/C371. All 23 enrolled participants completed all baseline study assessments. Compliance with the schedule of assessments ranged from 100% to a minimum of 64.7% at visit 6, for participants receiving cisplatin (Fig. 1). Average compliance with monitoring assessments across visits was 86.5% for tablet-based out-of-booth pure-tone audiometry, 84.8% for DPOAEs, and 84.3% for serum biomarkers. All visits were completed within 1 day before or after each chemotherapy cycle, apart from 3 participants who had their study visits at 4, 6, and 18 days after the end of the third chemotherapy cycle.

Across 13 participants, 28 reasons for noncompliance with the scheduled study visits and assessments were recorded. These included (i) patient-related factors in 12 (eg, too unwell for testing, hospital admission, conflicting hospital appointment, and shielding for COVID), (ii) organizational reasons in 9 (eg, research audiologist unavailable out of hours and change in chemotherapy schedule), and (iii) protocol deviations in 7 (eg, test data missing and audiometric testing equipment not charged).

### Pure-tone audiometry

**Figure 2:**
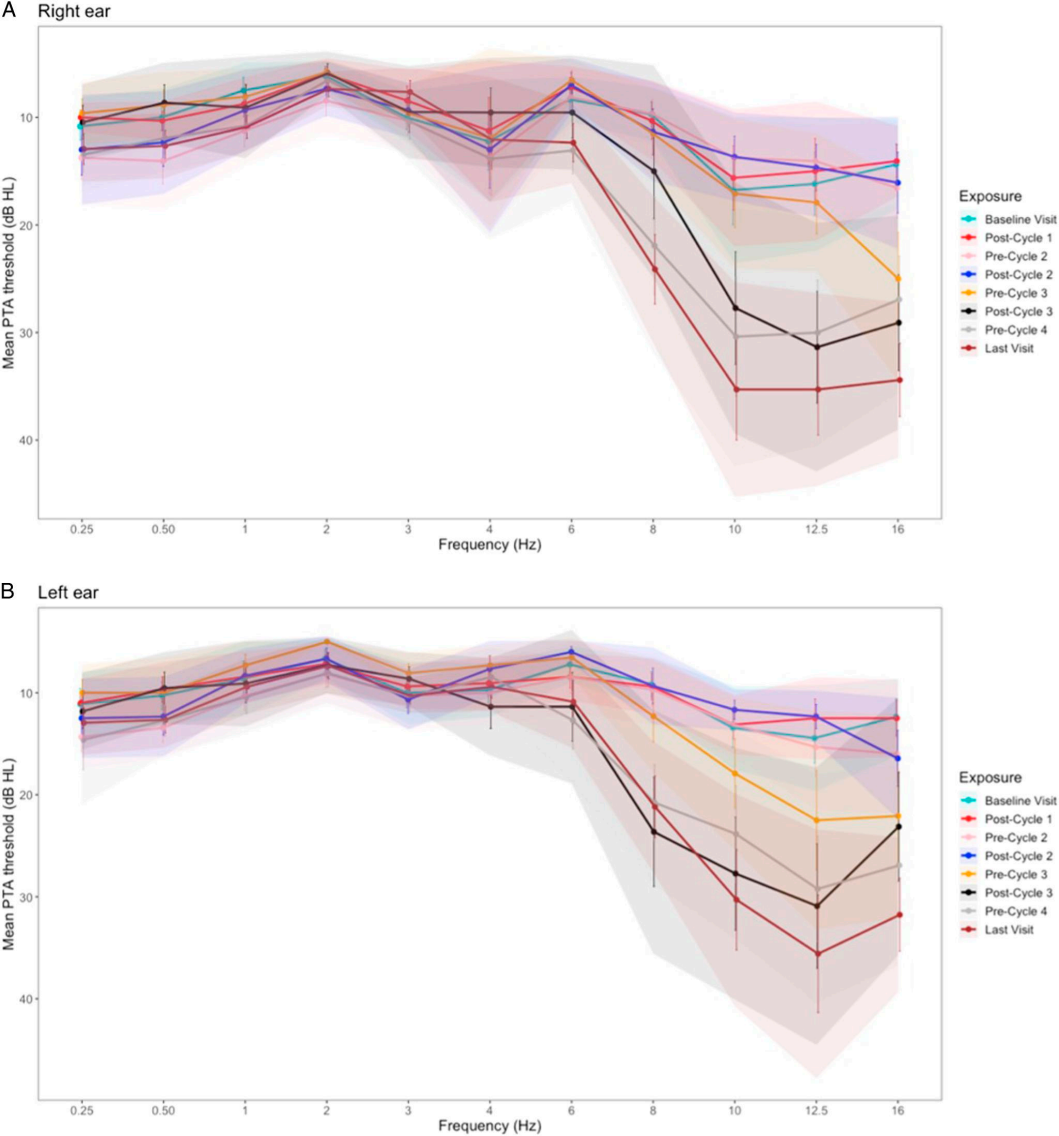
Mean pure-tone audiometry threshold at baseline and pre-/postadministration of cisplatin treatment, for the *A*, right and *B*, left ear. Error bars show 1 SE, and the shaded area shows the 95% CIs.

The 4 participants who were treated with carboplatin had no significant changes in their pure-tone audiometry thresholds from baseline to any of the follow-up assessments (eFigure 2, Supplemental Digital Content 2, http://links.lww.com/MAO/C372, for mean pure-tone audiometry thresholds for [a] right, and [b] left ear). The mean pure-tone audiometry thresholds (0.25 to 16 kHz) for the participants who received 4 cycles of cisplatin treatment regimen and demonstrated ototoxicity^34^ are provided in Figure 2. Changes in pure-tone audiometry thresholds for each individual participant were also recorded (eFigure 3, Supplemental Digital Content 3, http://links.lww.com/MAO/C373, for threshold changes for each participant, for [a] right, and [b] left ear).

### Otoacoustic emissions amplitudes

**Figure 3:**
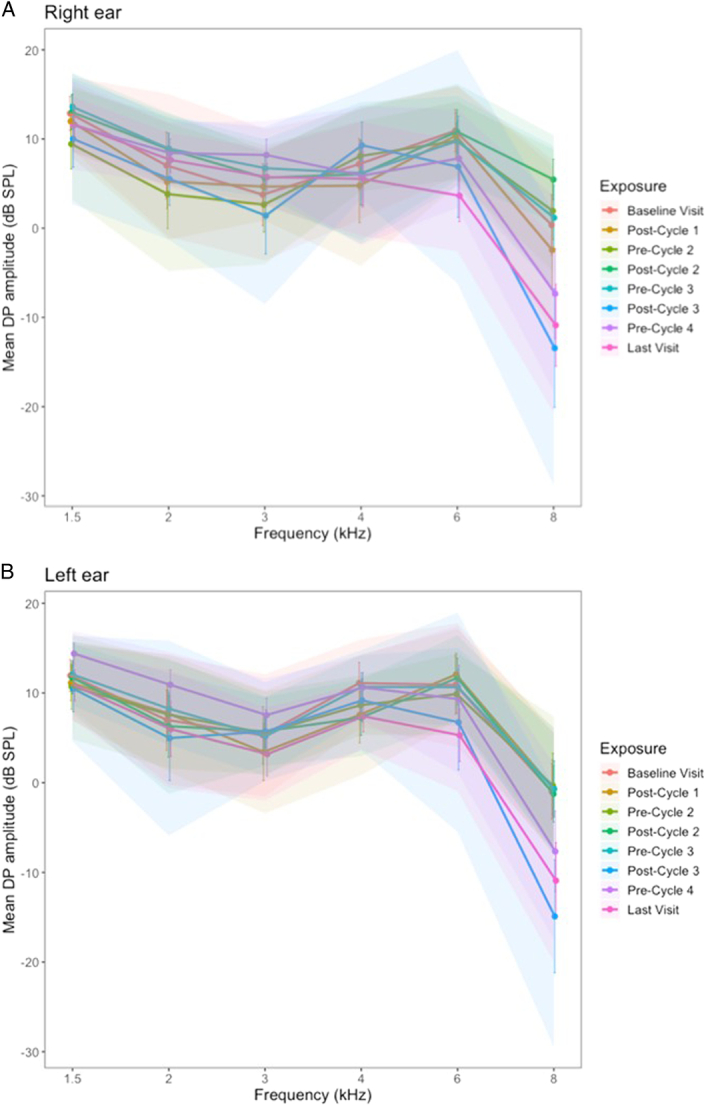
Mean distortion product amplitude at baseline and pre-/postadministration of cisplatin treatment, for the *A*, right and *B*, left ear. Error bars show 1 SE, and the shaded area shows the 95% CIs.

**Figure 4:**
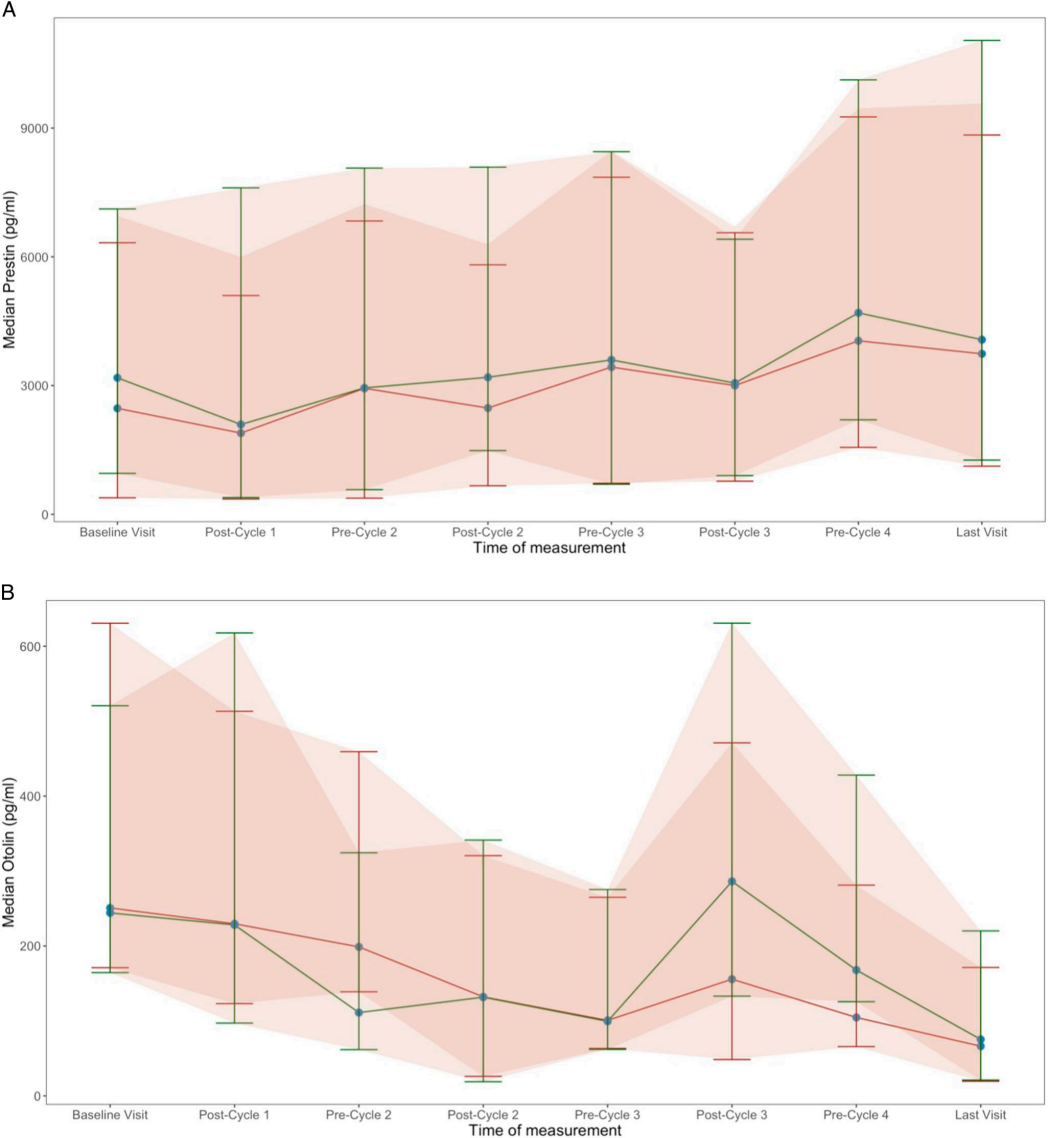
Median (*A*) serum prestin and (*B*) otolin-1 levels at baseline and pre-/postadministration of cisplatin chemotherapy treatment. The red line corresponds to the whole sample (n=18). The green line corresponds to the 10 participants showing PTA threshold compatible with ototoxicity. Error bars show the interquartile ratio and shaded area the 95% CIs.

The mean distortion product amplitude for the 18 participants who received 4 cycles of cisplatin-based treatment is provided in Figures 3 and 4.

### Patient-reported outcome measures

At baseline, all 23 participants completed an age-appropriate hearing health-related quality of life instrument (n=9 HEAR-28^30^, n=14 HHIA^31^), and 21 (91.3%) completed the TFI^32^. At the end of treatment, 19 (82.6%) participants completed an age-appropriate hearing health-related quality of life instrument (n=8 HEAR-28^30^, n=11 HHIA^35^), and 20 (87.0%) completed the TFI^32^.

There was no significant change in the scores for hearing health-related quality of life when compared with baseline. For HEAR-28^30^, the median change in scores was 1 (range: −6 to 13), and for HHIA^35^ it was 0 (range: 0 to 8). For the TFI^32^, the median change in score was 5 (range: −14 to 43); a significant worsening of scores was noted for 6 (33.3%) out of the 18 participants who completed both parts. Of those 6, 4 also had a significant deterioration in pure-tone hearing thresholds noted during their treatment.

Reasons for not completing the questionnaires at the end of treatment included feeling too tired, nauseous, or unwell; having no subjective tinnitus, therefore did not wish to complete the TFI32; and discontinuation of the original treatment regimen. One participant completed the HEAR-28^30^ at baseline, but by the end of treatment, no longer fitted the validated age criteria for this measure; and 1 participant had deceased.

### Observation of serum prestin and otolin-1 levels

Blood samples were collected, when possible, in a time window of 24 hours before or after the end of each treatment cycle for the 18 participants recruited at the UCLH, over the 8 visits. From the total of 126 patient serum samples taken, prestin levels were available for 126 and otolin-1 levels for 122. Otolin-1 levels were below the lower limit of quantification range of the ELISA kit for 4 samples. Audiometric changes indicative of ototoxicity^34^ were noted in 18 (78.2%) of the recruited participants. No trends were observed for otolin-1. Prestin levels tended to be higher in those who developed ototoxicity. Our sample size was insufficient to better define this observation. Furthermore, in the absence of a non-platinum comparator group, observed biomarker changes cannot be confidently ascribed to cisplatin ototoxicity, but rather to confounders. Therefore, the interpretability and validity of our biomarker observations are limited. Data from each participant, and the mean across participants over time, are provided in eFigure 4, Supplemental Digital Content 4, http://links.lww.com/MAO/C374 and eFigure 5, Supplemental Digital Content 5, http://links.lww.com/MAO/C375, for prestin and otolin-1, respectively.

## Discussion

### Key findings

Our study investigated the feasibility of monitoring ototoxicity in teenagers and young adults receiving treatment in an NHS cancer ambulatory care setting. We demonstrated a high level of recruitment (>67% of those eligible recruited) and compliance of more than 80% to scheduled serial tablet-based audiometry, DPOAE, and serum biomarkers testing. In our small cohort, prevalence of ototoxicity^34^ associated with cisplatin and/or carboplatin exposure was in keeping with global estimates^1^. Serum prestin levels tended to be higher in those who developed ototoxicity compared with those who did not, which warrants further research.

### Interpretation

Monitoring of ototoxicity in cancer patients is challenging, given the onerous chemotherapy schedules and the need to coordinate care with hearing specialists at frequently distant sites furnished with soundproof audiology booths. With several novel otoprotective drugs in the pipeline^15–17^, the first approved for clinical use^18–20^, there is an urgent need for new approaches to real-time ototoxicity monitoring in patients undergoing platinum-based chemotherapy. Randomized controlled trials investigating the role of otoprotective drugs^36^ report audiometric compliance rates ranging from 70% to 90%, whereas in clinical practice, the rates are reported as low as 5%^37^. Bedside tablet-based audiometry reduces the organizational burden of additional hospital appointments in frequently complex chemotherapy schedules, with consequent potential improvement in patient experience and earlier detection of ototoxicity.

Currently available tablet-based audiometers have demonstrated 80.1% to 95.8% accuracy within 10 dB of a standard clinical audiometer^10,38,39^; and may provide a reasonable alternative to standard in-booth audiometry for this population, who may be unwell, undergoing intense medical treatment and requiring frequent repeat hearing tests. However, this will need to be balanced against the availability of a mobile audiology workforce that is able to supervise such testing. Noncompliance with scheduled testing in our study commonly occurred because of organizational issues: cycles of chemotherapy are frequently rescheduled at short notice for clinical reasons, and it was not always possible to have an audiologist available to coincide with the end of treatment (particularly late evenings or weekends).

Integrating the patient perspective into cancer care can improve patient outcomes and health resource utilization^40^. In our study cohort, it proved feasible for both teenagers and adult participants to complete the selected quality of life (HEAR-28^30^, HHIA^35^) and tinnitus (TFI^32^) patient-reported outcome measures. Clinically significant change in scores was only noted on the TFI^32^.

Finally, this study explored the hypothesis that baseline serum prestin and otolin-1 levels would be altered in relation to PTA threshold shifts. We observed these biomarker changes in participants with ototoxicity and those with no documented ototoxicity separately. One of the key strengths of blood biomarker monitoring is the provision of a noninvasive and objective method to assess cochlear health, probably even before clinical symptoms manifest. Unlike traditional audiometric methods, biomarker measurement can be easily integrated into routine blood tests during oncology treatment, minimizing the need for additional appointments or specialized equipment and personnel. In our exploratory observations, the subgroup of patients with ototoxicity (10 out of 18 participants who received 4 cycles of cisplatin) exhibited higher levels of prestin. This observation is interesting; however, it remains descriptive and does not support firm conclusions about correlations or clinical utility. Further research is warranted to validate the specificity and sensitivity of prestin as a reliable ototoxicity indicator and to establish its utility in clinical practice.

### Limitations

This observational study was conducted across 2 large university hospitals, which are both tertiary cancer and audiology centers. These sites are not representative of smaller district hospitals with potentially fewer research support staff. Future studies should consider evaluating the feasibility and scalability across a broader range of clinical contexts. Nonetheless, self-test modes are available on most tablet-based audiometers, which have had varying degrees of validation in adults, and could be considered for further evaluation at such sites. This may also help reduce noncompliance with the testing schedule, since one of the key causes of noncompliance was the research audiologist not being available at the time of the end of chemotherapy cycles.

While our study has observed the potential relationship between ototoxicity, prestin, and otolin-1, considerable further work is needed to establish the role of serum biomarkers in ototoxicity. Currently, there are no standardized thresholds for levels indicative of ototoxicity; the potential influence of confounding factors, such as individual variability in biomarker expression, is unknown, and concerns remain regarding the quality and specificity of commercial ELISA kits available for measuring prestin^41^. No comparator control group of cancer patients not exposed to platinum-based agents was available in this study. Larger, longitudinal studies with appropriate control groups will be required to determine the clinical utility and normative variability of prestin, otolin-1, and other candidate biomarkers in this context.

### Generalizability

This study has shown that tablet-based audiometry and serial biomarker blood testing are feasible within a cancer ambulatory care setting. While it is interesting to observe that prestin levels tended to be higher in those who developed ototoxicity, further research is required to establish biomarker utility in clinical practice. Commonly reported barriers to implementing ototoxicity monitoring for patients undergoing platinum-based chemotherapies include lack of integrated interdisciplinary care, referral issues, and insufficient resources^9^. Tablet-based assessment and monitoring within an oncology center could be a feasible solution for adolescents and young adults in the future.

## Conclusions

In a research setting, high compliance with ototoxicity monitoring with tablet-based audiometry, mobile DPOAE testing, and serum biomarker analysis can be achieved within cancer ambulatory care. This study offers key learnings for upcoming trials of novel otoprotective drugs in patients undergoing chemotherapy.

## Acknowledgments

The authors thank the patients and families who gave their time and support for this study during challenging times. They also thank the evidENT team at the UCL Ear Institute for their support in study coordination, study delivery, data management, and data analysis: Khadija Ahmed, Bebela Kalala, Fei Long, Sidal Matyar, Catherine Pengelly, and Zahrah Uddin. They are grateful to Professor John Hartley, Victoria Spanswick, Arran Speirs, Nas Mahmoud, Sharyar Hadi, Yashma Pathak, Angeliki Karamani, Riaz Jannoo, Ridesh Rai, Megan Driscoll, and Sonnika Lamont of the UCL ECMC GCLP Facility for their support in processing and storage of blood samples.

## Supplementary Material
