# Supplementary material for: Early Detection of Ototoxicity Using Serial Mobile Audiometry, Otoacoustic Emissions Testing, and Inner Ear Biomarker Measurement in Patients Receiving Platinum-Based Chemotherapy Treatment: It is Feasible to Implement in a National Health Service (NHS) Cancer Ambulatory Care Setting
Source: Otol Neurotol. 2026 Feb 25;47(4):539–48. doi: 10.1097/MAO.0000000000004856 (PMC12970545; doi:10.1097/MAO.0000000000004856)

**SUPPLEMENTAL DIGITAL CONTENT 1**

**eFigure 1.** Summary of schedule of assessments including screening and enrolment of eligible participants, and procedures conducted at each study visit for those receiving **(a)** cisplatin, and **(b)** carboplatin chemotherapy, as per study protocol.

1. Cisplatin chemotherapy regimen schedule.


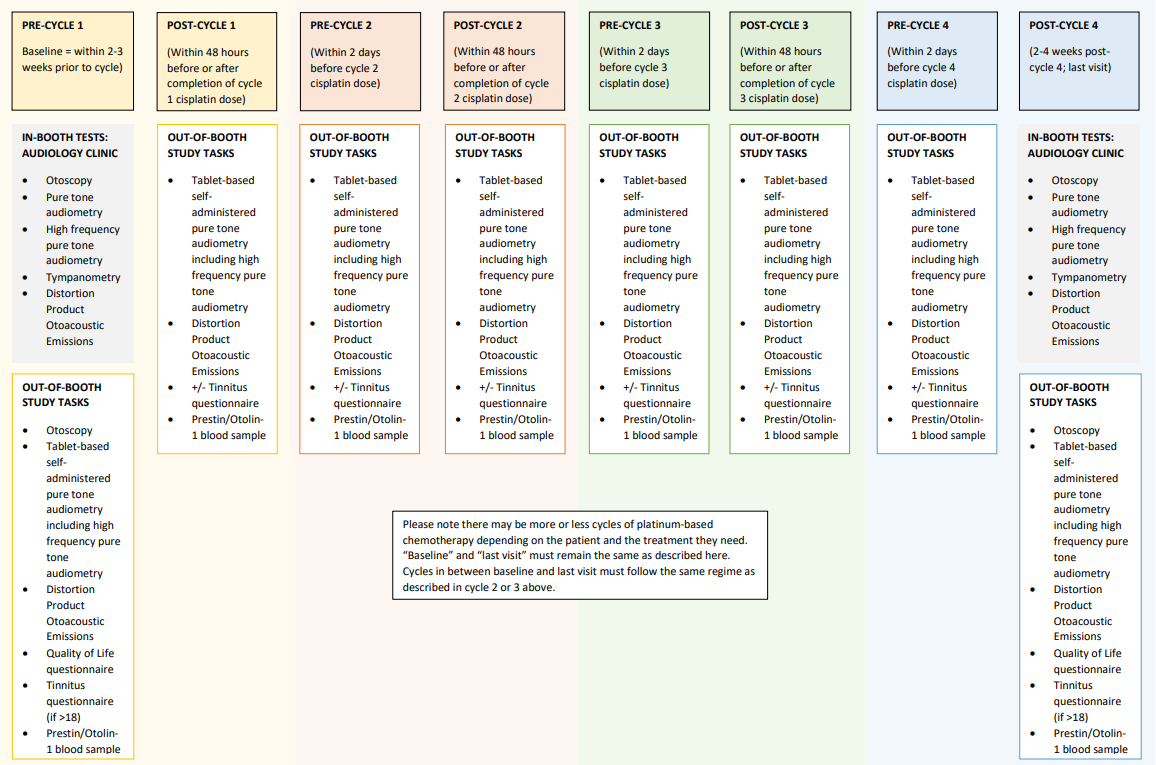


1. Carboplatin chemotherapy regimen schedule.


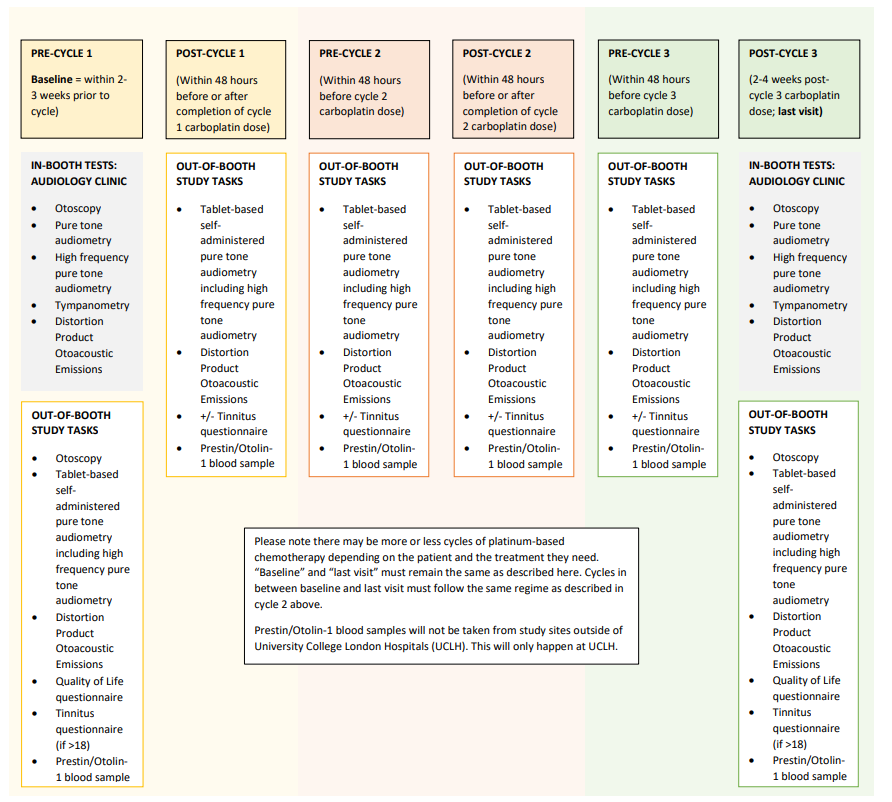

Supplement: Supplementary file 1 [file mao-47-539-s001.docx]
