# Supplementary material for: Early Detection of Ototoxicity Using Serial Mobile Audiometry, Otoacoustic Emissions Testing, and Inner Ear Biomarker Measurement in Patients Receiving Platinum-Based Chemotherapy Treatment: It is Feasible to Implement in a National Health Service (NHS) Cancer Ambulatory Care Setting
Source: Otol Neurotol. 2026 Feb 25;47(4):539–48. doi: 10.1097/MAO.0000000000004856 (PMC12970545; doi:10.1097/MAO.0000000000004856)

**SUPPLEMENTAL DIGITAL CONTENT 2**

**eFigure 2.** Mean pure tone audiometry threshold shifts (0.25-16 kHz) for n=4 participants after exposure to carboplatin, for the **(a)** right and **(b)** left ear. Error bars show 1 standard error and shadow area the 95% confidence intervals.

**(a)**


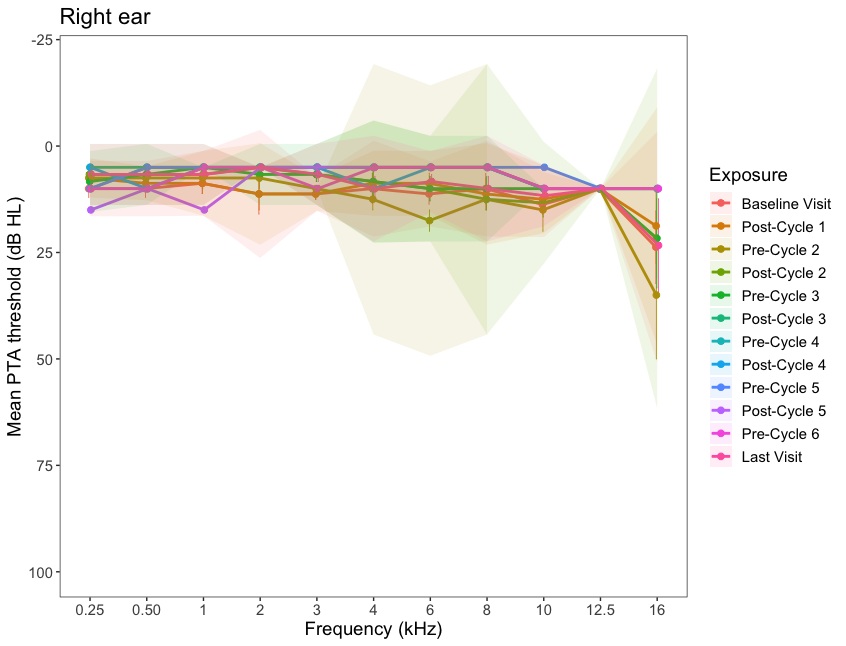


**(b)**


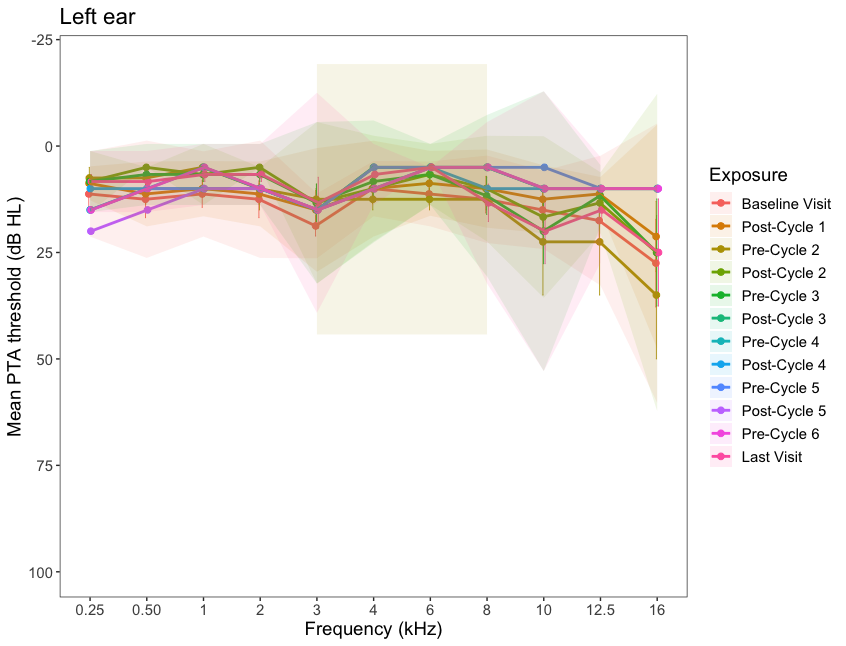

Supplement: Supplementary file 2 [file mao-47-539-s002.docx]
