# Supplementary material for: Early Detection of Ototoxicity Using Serial Mobile Audiometry, Otoacoustic Emissions Testing, and Inner Ear Biomarker Measurement in Patients Receiving Platinum-Based Chemotherapy Treatment: It is Feasible to Implement in a National Health Service (NHS) Cancer Ambulatory Care Setting
Source: Otol Neurotol. 2026 Feb 25;47(4):539–48. doi: 10.1097/MAO.0000000000004856 (PMC12970545; doi:10.1097/MAO.0000000000004856)

**SUPPLEMENTAL DIGITAL CONTENT 3**

**eFigure 3.** Air-conduction pure tone audiometry thresholds (0.25-16 kHz) for **(a)** right and **(b)** left ear, for each participant. Only participants having received cisplatin chemotherapy regimen are included. Participants Oto-3, Oto-4, Oto-10, Oto-15, Oto-20, and Oto-21 developed bothersome tinnitus by the last visit. Participants Oto-02, Oto-04, Oto-06, Oto-09, Oto-11, Oto-15, Oto-17, Oto-19, Oto-20, Oto-21 suffered ototoxicity^36^.

**(a)**


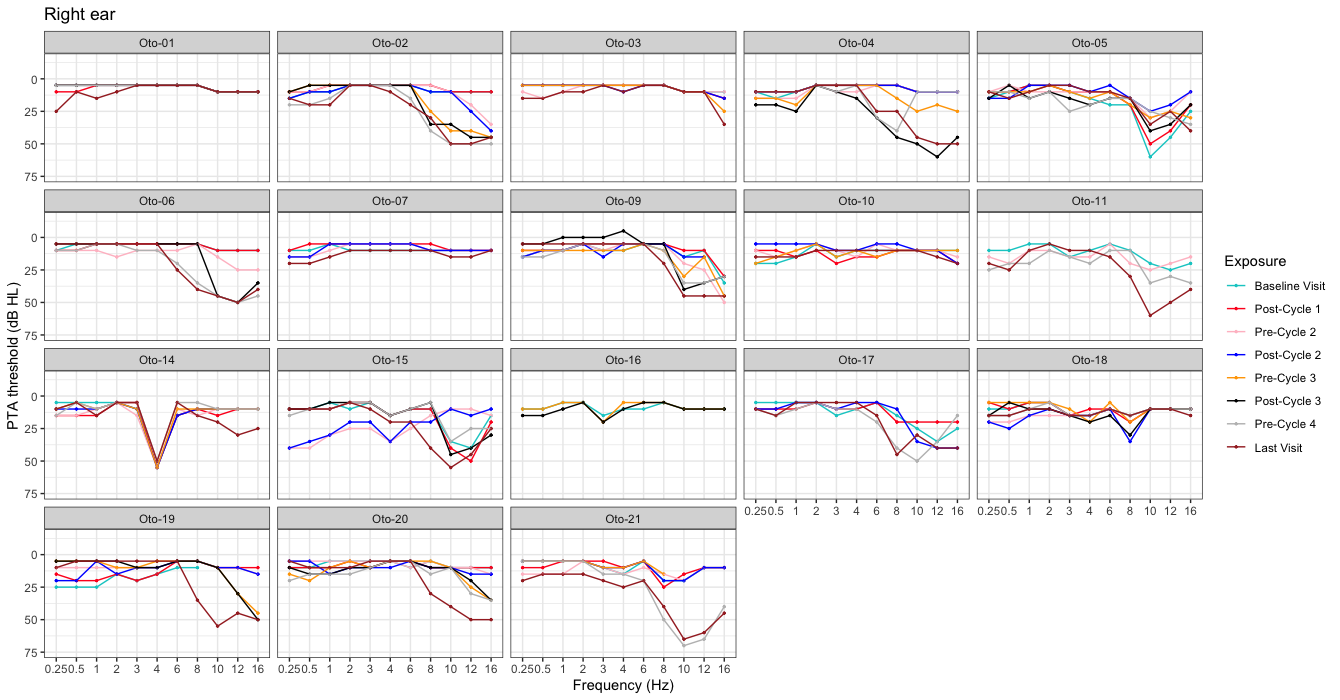


**(b)**


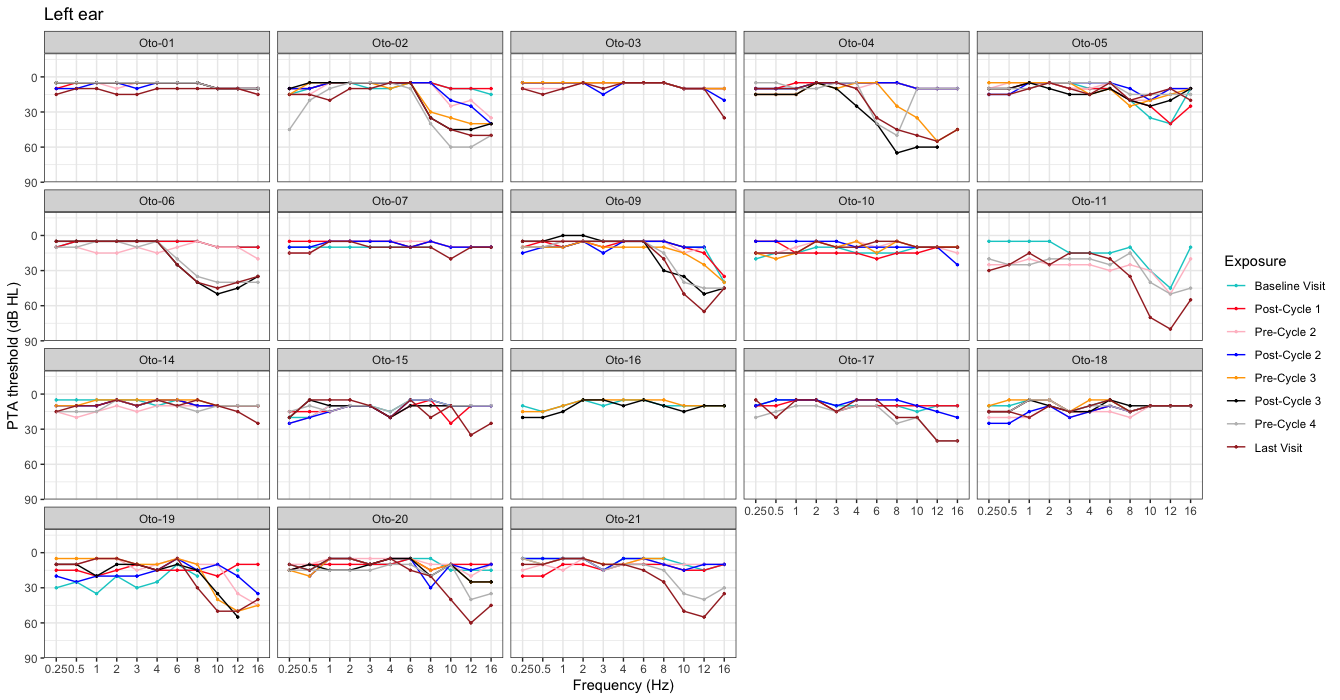

Supplement: Supplementary file 3 [file mao-47-539-s003.docx]
