# Supplementary material for: Early Detection of Ototoxicity Using Serial Mobile Audiometry, Otoacoustic Emissions Testing, and Inner Ear Biomarker Measurement in Patients Receiving Platinum-Based Chemotherapy Treatment: It is Feasible to Implement in a National Health Service (NHS) Cancer Ambulatory Care Setting
Source: Otol Neurotol. 2026 Feb 25;47(4):539–48. doi: 10.1097/MAO.0000000000004856 (PMC12970545; doi:10.1097/MAO.0000000000004856)
Supplement: Supplementary file 4 [file mao-47-539-s004.docx]

**SUPPLEMENTAL DIGITAL CONTENT 4**

**eFigure 4.** Serum prestin levels (pg/ml) for each individual participant (n=18), and the mean across participants over time. The X axis corresponds to the different measurement time points, while Y axis corresponds to the serum prestin levels in pg/ml. The table provides the summary statistics for prestin levels in each measurement time point.


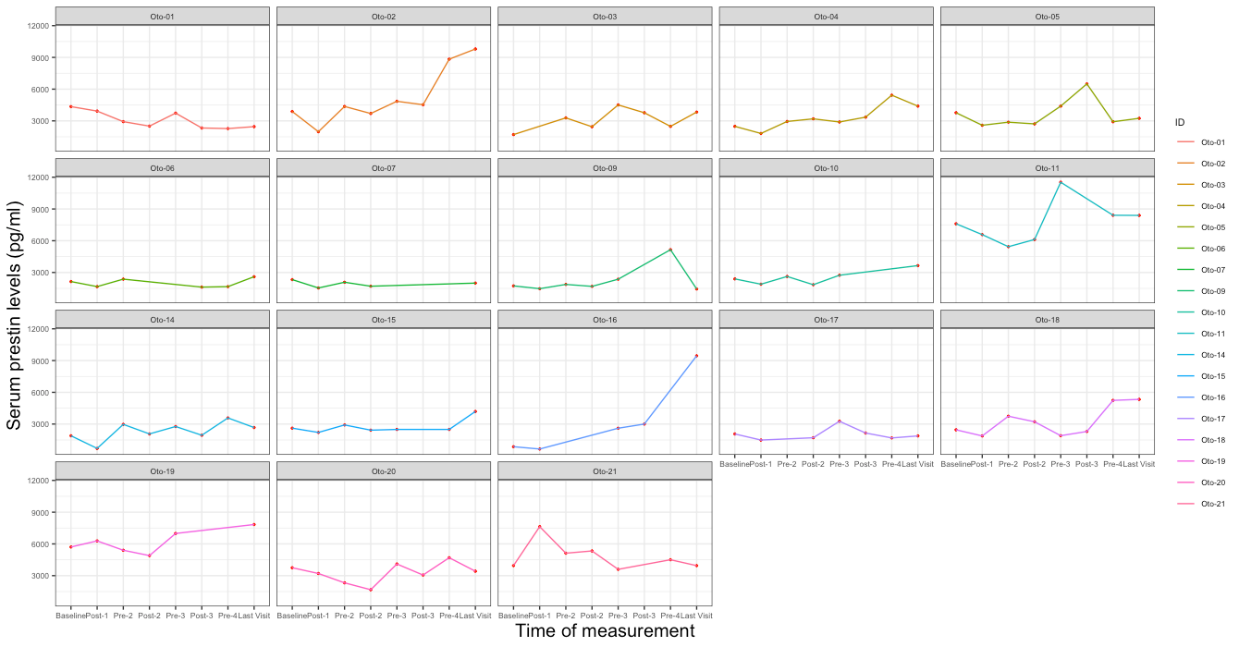


| **Exposure** | **n** | **min** | **max** | **median** | **mean** | **SD** | **SE** | **CI** |
| --- | --- | --- | --- | --- | --- | --- | --- | --- |
| **Baseline visit** | 18 | 861.960 | 7603.389 | 2470.428 | 3094.299 | 1631.593 | 384.570 | 811.372 |
| **Post-Cycle 1** | 17 | 642.706 | 7633.861 | 1893.000 | 2793.492 | 2097.077 | 508.616 | 1078.218 |
| **Pre-Cycle 2** | 16 | 1871.400 | 5436.168 | 2936.450 | 3328.566 | 1158.627 | 289.657 | 617.389 |
| **Post-Cycle 2** | 16 | 1672.166 | 6114.784 | 2477.224 | 2952.467 | 1397.005 | 349.251 | 744.412 |
| **Pre-Cycle 3** | 16 | 1902.521 | 11528.607 | 3431.259 | 4046.577 | 2353.711 | 588.428 | 1254.204 |
| **Post-Cycle 3** | 11 | 1617.789 | 6499.322 | 3000.905 | 3141.457 | 1405.515 | 423.779 | 944.238 |
| **Pre-Cycle 4** | 14 | 1664.549 | 8829.943 | 4043.418 | 4239.830 | 2274.972 | 608.012 | 1313.530 |
| **Last visit** | 18 | 1441.366 | 9792.934 | 3740.572 | 4473.685 | 2627.845 | 619.389 | 1306.796 |

(n: number, min: minimum, max: maximum, SD: standard deviation, SE: standard error, CI: confidence interval)
