# Supplementary material for: Early Detection of Ototoxicity Using Serial Mobile Audiometry, Otoacoustic Emissions Testing, and Inner Ear Biomarker Measurement in Patients Receiving Platinum-Based Chemotherapy Treatment: It is Feasible to Implement in a National Health Service (NHS) Cancer Ambulatory Care Setting
Source: Otol Neurotol. 2026 Feb 25;47(4):539–48. doi: 10.1097/MAO.0000000000004856 (PMC12970545; doi:10.1097/MAO.0000000000004856)
Supplement: Supplementary file 5 [file mao-47-539-s005.docx]

**SUPPLEMENTAL DIGITAL CONTENT 5**

**eFigure 5.** Otolin-1 levels (pg/ml) for each individual participant (n=18), and the mean across participants over time. The X axis corresponds to the different measurement time points, while Y axis corresponds to the serum otolin-1 levels in pg/ml. The table provides the summary statistics for otolin-1 levels in each measurement time point.


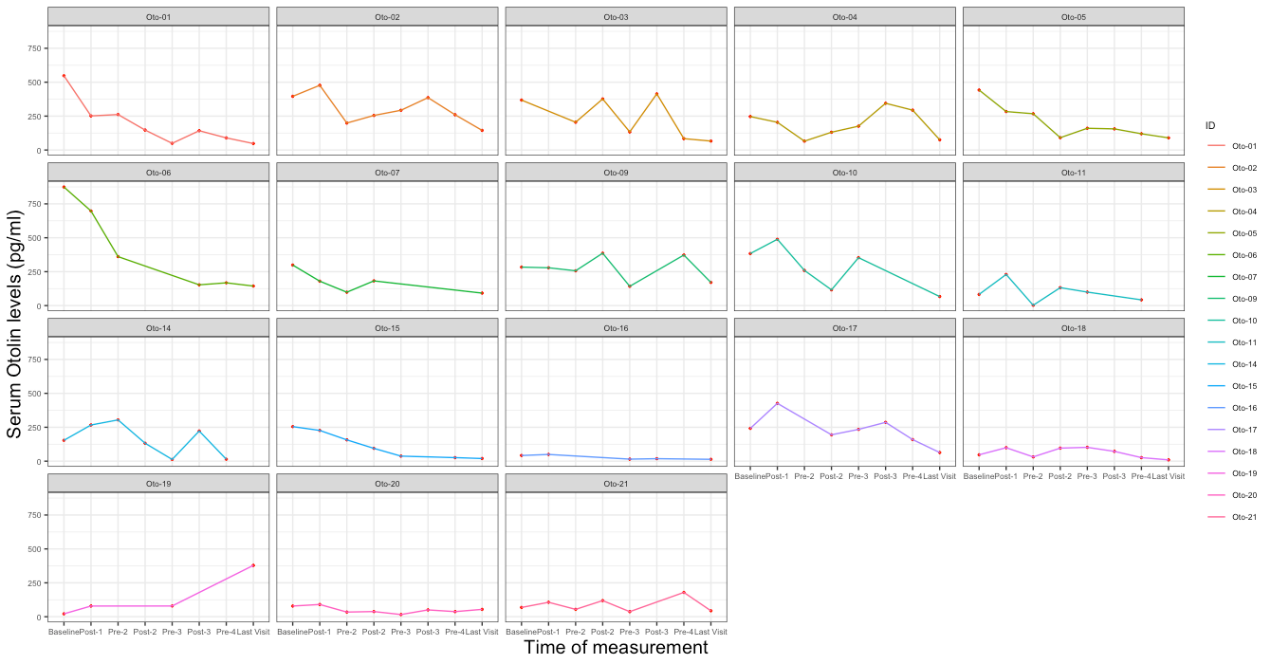


| **Exposure** | **n** | **min** | **max** | **median** | **mean** | **SD** | **SE** | **CI** |
| --- | --- | --- | --- | --- | --- | --- | --- | --- |
| **Baseline visit** | **18** | 21.579 | 874.400 | 250.744 | 268.404 | 217.278 | 51.213 | 108.05 |
| **Post-Cycle 1** | **17** | 49.562 | 697.500 | 229.663 | 260.920 | 174.382 | 42.294 | 89.659 |
| **Pre-Cycle 2** | **15** | 2.544 | 361.100 | 198.800 | 170.522 | 114.994 | 29.691 | 63.682 |
| **Post-Cycle 2** | **15** | 38.254 | 386.300 | 132.137 | 166.137 | 100.845 | 26.038 | 55.846 |
| **Pre-Cycle 3** | **16** | 12.647 | 353.500 | 100.728 | 121.299 | 102.412 | 25.603 | 54.572 |
| **Post-Cycle 3** | **11** | 18.151 | 414.100 | 155.700 | 204.093 | 137.276 | 41.390 | 92.223 |
| **Pre-Cycle 4** | **14** | 14.254 | 372.700 | 104.650 | 133.767 | 111.747 | 29.866 | 64.521 |
| **Last visit** | **16** | 9.394 | 378.626 | 66.2900 | 92.497 | 89.499 | 22.375 | 47.690 |

(n: number, min: minimum, max: maximum, SD: standard deviation, SE: standard error, CI: confidence interval)
